# Metabolic Syndrome, Gastric Cancer Mortality and Competing Risk Survival Analysis

**DOI:** 10.1016/j.ebiom.2016.12.015

**Published:** 2016-12-23

**Authors:** Mohamad Amin Pourhoseingholi, Sara Ashtari, Nastaran Hajizadeh, Mohammad Reza Zali

**Affiliations:** a Gastroenterology and Liver Diseases Research Center, Research Institute for Gastroenterology and Liver Diseases, Shahid Beheshti University of Medical Sciences, Tehran, Iran.; b Basic and Molecular Epidemiology of Gastrointestinal Disorders Research Center, Research Institute for Gastroenterology and Liver Diseases, Shahid Beheshti University of Medical Sciences, Tehran, Iran.

Hu and colleagues in their recently published paper in *EBioMedicine* (Hu et al., 2017-in this issue) regarding the impact of metabolic syndrome (Mets) on gastric cancer mortality, conducted a prospective study (Fujian prospective investigation of cancer) on 3012 patients with gastric cancer, from 2000 to 2010, which latest follow-up have been done in 2015. During these 15 years, 1331 of 3012 patients died of gastric cancer. The median survival time (MST) of patients with MetS was 31.3 months, which was significantly shorter than that of MetS-free patients (157.1 months) and the coexistence of MetS before surgery was associated with a 2.3-fold increased risk for gastric cancer mortality. Also in a multivariate analysis, they reported the increasing hazard ratio of gastric cancer mortality, associated with invasion depth T1/T2, regional lymph node metastasis N0, positive distant metastasis, TNM stage I/II, intestinal type, negative tumor embolus and tumor size ≤ 4.5 cm. finally, survival tree analysis confirmed the top splitting role of TNM stage, followed by MetS or hyperglycemia with remarkable discrimination ability.

This study has a strong methodology, since it employed the FIESTA study (Peng et al., 2016), initiated in January 2000 as an ongoing prospective cohort of common digestive system tumors, including esophageal cancer, gastric cancer and colorectal cancer. Besides, the follow-up process, data gathering methods, MetS assessment and Demographic and Clinicopathologic Characteristics which included in the analysis, were the other positive points of this study.

Also, for statistical analysis, multivariate Weibull proportional hazards regression model before and after adjusting for confounding factors, were used.

Gastric cancer is one of the most common cancers in the world. It is rarely detected early, and the prognosis remains poor (Pourhoseingholi et al., 2009) and recent study indicated association between MetS and increasing risk of gastric adenocarcinoma (Lin et al., 2015). The main finding of Hu and colleagues is an important issue, not only for clinicians who are facing with the gastric cancer patients, but also for health policy makers in Asian countries who are challenging with the burden of cancers in their own populations. We know that, the burden of gastrointestinal cancer is increasing in Asia because of aging, growth of the population and the risk factors including smoking, obesity, and changing lifestyle (Pourhoseingholi et al., 2015), which would increases the risk of MetS too. Controlling the MetS is a potential key to controlling the cancer burden in Asian population. Although this study has a long period of follow-up and the number of patients under study is considerable, the number of patients who died from other cause of gastric cancer and eliminated from the analysis is crucial even thought there was no significant differences according to demographic or clinical factors between these two groups. 235 patients died of causes other than gastric cancer, vs. 1331 patients died of gastric cancer. Since MetS increases cardiovascular disease morbidity and mortality (Katzmarzyk et al., 2006) and predicts long-term mortality in subjects without established diabetes mellitus (Won et al., 2016), the assumption of independence between these eliminated mortality and MethS is skeptical and there would be a potential under estimation of hazard rate of gastric cancer mortality, corresponding to these competing risk of death. When competing events are present in survival analysis, Cox regression and Kaplan–Meier method are invalid, because a subject who has failed in other competing risks is treated as a censored subject. So alternative methods specifically designed for analyzing competing risks data, which are developed for parametric models could be employed to estimate the true hazard rate (Baghestani et al., 2016). For further study, considering these models, instead of omitting the data would be beneficial to understand the better rule of MetS on any gastrointestinal cancer mortalities.

## Disclosure

The authors declared no conflicts of interest.
